# Survival Outcomes among Human Epidermal Growth Factor Receptor 2- (HER2-) Positive Breast Cancer Patients at Kenyatta National Hospital

**DOI:** 10.1155/2021/3115727

**Published:** 2021-12-15

**Authors:** Gloria Tuwei, Amsalu Degu

**Affiliations:** Department of Pharmaceutics and Pharmacy Practice, School of Pharmacy and Health Sciences, United States International University-Africa, Nairobi, Kenya

## Abstract

**Introduction:**

HER2-positive breast cancer is associated with poor outcomes and higher mortality rates than other breast cancer subtypes. The advent of trastuzumab has significantly changed the natural history of HER2-positive breast cancer. However, it is not an affordable treatment option in sub-Saharan African countries. Because of the expense, most patients in our setting do not receive trastuzumab for the optimal control of their disease. Additionally, there is a lack of comprehensive data about the survival outcomes of HER2-positive breast cancer patients in our setting. The present study was aimed at determining the survival outcomes among HER2-positive breast cancer patients at the Oncology Department of Kenyatta National Hospital.

**Methods:**

A hospital-based retrospective cohort design was used to evaluate the survival outcomes among patients with HER2-positive breast cancer treated from 1^st^ January 2015 to 31^st^ December 2019 at Kenyatta National Hospital. A total of 50 eligible HER2-positive breast cancer patients were included in the study. In the predesigned data abstraction tool, data were collected by reviewing the medical records of the patients. The data were entered and analyzed using the Statistical Package for the Social Sciences version 27 software. The mean survival time was estimated using Kaplan-Meier survival analysis.

**Results:**

The mean age was 45.44 ± 12.218 years, with a majority (80%) of the patients being below 60 years. Most patients (64%) had advanced-stage disease. The median follow-up time for patients with curative stages of breast cancer was 41 months, while the median follow-up time for those with the advanced incurable disease was 8.5 months. The 4-year survival rate was 62.5% for those curable-stage HER2-positive breast cancer compared to 5.6% for those with metastatic disease at presentation.

**Conclusion:**

The 4-year survival rate for both early-stage and advanced-stage HER2-positive breast cancer in our setting is suboptimal when compared to existing outcome data from health care systems where trastuzumab is more widely available.

## 1. Introduction

Cancer is a combination of diseases distinguished by unlimited growth and spread of malignant cells [1]. Among the many forms of cancer diagnosed globally, breast cancer is the primary type of cancer that affects most women [2]. Breast cancer is a significant public health concern globally, accounting for 1,960,682 new cases and 611,625 deaths [3].

Cancer is becoming an issue of concern for many Kenyans today, with its prevalence rising rapidly over the past few years. It is the third major cause of death after infectious and cardiovascular diseases in Kenya [4]. In 2020, breast cancer accounted for 16.1% of total cancer cases in Kenya [5].

HER2-positive breast cancer is a highly aggressive, fast-growing type of cancer that accounts for about 20% of breast cancers [6]. Prior to the advent of HER2-directed therapies such as trastuzumab, HER2-positive breast cancer was associated with poorer outcomes and higher mortality rates than other breast cancer subtypes [7, 8]. The advent of trastuzumab, as well as other HER2-directed therapies, has significantly changed the treatment paradigm for patients with HER2-positive disease and dramatically improved outcomes [9].

Yamashiro et al. reported that the five- and ten-year overall survival rates were 96% and 92.7%, respectively, among HER2-positive breast cancer patients with early-stage disease after treatment including trastuzumab [10]. A systematic review reported a significant improvement in the overall survival among trastuzumab-treated HER2-positive breast cancer patients in the modern era with the survival outcomes that are somewhat better compared to the other breast cancer subtypes [11]. A study in the United States reported that the four-year survival rate among HER2-positive breast cancer patients was 90.3% [12].

Although trastuzumab is a frontline adjuvant treatment option in combination with chemotherapy in HER2-positive breast cancer patients that achieves better outcomes, it is not an affordable treatment option in sub-Saharan African countries [13]. Because of the expensive nature of trastuzumab treatment, most patients in our setting did not get this treatment for the optimal control of their disease. There is also a lack of comprehensive data about the survival outcomes of HER2-positive breast cancer patients in our setting. The present study was aimed at determining the survival outcomes and associated factors among HER2-positive breast cancer patients at the Oncology Department of Kenyatta National Hospital (KNH).

## 2. Methods

### 2.1. Study Design, Setting, and Period

A retrospective cohort study design was employed among HER2-positive breast cancer patients treated at the Kenyatta National Hospital (KNH) from 1^st^ January 2015 to 31^st^ December 2019. The hospital is located in Nairobi, Kenya, and is the oldest and largest public referral hospital in Kenya. It was founded in 1901 as the Native Civil Hospital, with a maximum bed capacity of about 40 patients. In 1952, the hospital was renamed the King George VI hospital and subsequently became the Kenyatta National Hospital in honor of Jomo Kenyatta, Kenya's first president.

### 2.2. Target Population

The medical records of all HER2-positive breast cancer patients admitted at the Oncology Department of KNH from 1^st^ January 2015 to 31^st^ December 2019 who fulfilled the inclusion criteria were involved in the study.

### 2.3. Eligibility Criteria

#### 2.3.1. Inclusion Criteria

- All adult patients 18 years and above with a confirmed diagnosis of HER2-positive breast cancer treated in the hospital from 1^st^ January 2015 to 31^st^ December 2019
- Patients with complete medical records of diagnosis, stage of cancer, and treatment regimen

#### 2.3.2. Exclusion Criteria

- Patients who had incomplete medical records
- Patients with their HER-2 status who had not been clearly defined
- Patients with unclear treatment regimens

### 2.4. Sample Size Determination

All HER2-positive breast cancer patients treated in the Oncology Department from 2015 to 2019 and fit the inclusion criteria were involved in the study. There were no formal sample size calculations due to the nature of this retrospective review, and a total of 50 eligible medical records of HER2-positive breast cancer patients were included in the study.

### 2.5. Research Instrument and Data Collection Techniques

A data abstraction tool was designed in reference to previous studies of the standard methods of reporting cancer treatment outcomes. The patients' records were obtained from the Health Information Department of Kenyatta National Hospital. Pertinent information such as patient's socio-demographic, clinical characteristics, the type of treatment given, the status of the patient in the last follow-up period, and survival time was recorded after reviewing the patients' medical records. Descriptive statistics were employed to assess mortality rates as well as median overall survival times and mean survival estimates.

### 2.6. Pilot Study

A pretest was conducted on 5% of the sample population one week before the actual start of the study to assess feasibility. Any required adjustments to the data abstraction tool were implemented before beginning the main study.

### 2.7. Study Variables

The dependent variables for this study were patients' survival outcomes. Demographic variables such as age, gender, level of education, occupation, and marital status and patient characteristics such as comorbidities, number of medications, histological type of breast cancer, stage of breast cancer, and treatment regimens were collected.

### 2.8. Data Analysis

Data were entered and analyzed using the Statistical Package for the Social Sciences version 27 software. The mean survival time was estimated using the Kaplan-Meier method.

## 3. Results

### 3.1. Socio-Demographic Characteristics

Socio-demographic characteristics of the study population are presented in Table 1. The mean age of the study participants was 45.4 ± 12.2 years, with the majority of patients being younger than 60 (*n* = 40, 80%). Among the 50 study participants, 35 (70%) were married, 7 (14%) were single, 7 (14%) were divorced, and 1 (2%) was widowed. Half of the study population had attained tertiary education, while 6 (12%) had only primary education. Housewives and private employees comprised 22% of the study population, while 2 (4%) were merchants. The majority (84%) of the study participants had no history of substance use, 7 (14%) had a history of alcohol use, and 1 (2%) was a cigarette smoker. Half of the study population (25, 50%) had a family history of cancer.

### 3.2. Clinical Characteristics of the HER2-Positive Breast Cancer Patients

Clinical characteristics of the study population are presented in Table 2. All study participants had invasive ductal carcinoma of the breast with 18 (36%) having stage IV disease at diagnosis. For those with earlier stage, potentially curable disease, 17 (34%) were stage II, 14 (28%) were stage III, and only one patient had stage I disease. Among the study participants, 22 (44%) had comorbidities, 16 (32%) with one and 6 (12%) had two comorbidities. The predominant comorbidity was diabetes mellitus (11, 22%), followed by hypertension (14%) and retroviral disease (14%), while the least prevalent was deep vein thrombosis (2, 4%) and anaemia (1, 2%).

### 3.3. Treatment Regimen Administered to the HER2-Positive Breast Cancer Patients

The treatment regimens administered to the study patients were radiotherapy, chemotherapy, surgery, and trastuzumab. Of the study patients, 34.4% and 61.1% of the curable- and incurable-stage patients have completed the planned chemotherapy, respectively (Table 3).

### 3.4. Survival Outcomes

The median follow-up time among curable-stage breast cancer was 41 months, while the median follow-up time for those with advanced-stage disease was 8.5 months. The median overall survival of curable-stage disease was 35 months (range: 10-56 months) and for those with advanced-stage disease was nine months (range: 4-18 months). The 4-year survival rate was 62.5% for those curable disease versus 5.6% for those with metastatic disease at presentation.

The mean survival estimate was 36.78 ± 15.5 months among curable breast cancer patients versus 8.89 ± 3.4 months among metastatic stage of HER2-positive breast cancer patients. Diabetes mellitus was the only comorbidity resulting in a statistically significant difference in mean survival estimates among early-stage curable breast cancer patients (log-rank *p* < 0.05) (Table 4).

## 4. Discussion

This study assessed the survival outcomes among 50 patients with HER2-positive breast cancer treated at the Kenyatta National Hospital. The 4-year survival rate was 62.5% for those curable-stage diseases. In contrast, a Canadian study revealed the overall survival of 88.6% among early-stage HER2-positive breast cancer patients [14]. Similarly, another study showed a higher overall survival (87.1%) among nonmetastatic HER-positive breast cancer patients [15]. The low survival rate in our setting is probably linked to differences in the level of care among study settings. Only a few of our study participants were treated with trastuzumab which likely contributed to the low survival rate observed in our setting.

The four-year survival rate for those with metastatic and incurable disease was only 5.6% which contrasts with an Algerian study where a 5-year survival rate of 38.8% was observed [16]. The mean survival time was 8.89 ± 3.4 months among our patients with metastatic disease and contrasts with that of Guo et al. who reported the median overall survival times of 46.2 months among advanced-stage HER2-positive breast cancer patients [17]. This low overall survival rate in our setting may be attributed to many factors, including the lack of early screening programs, a higher proportion of advanced-stage cancer at the time of diagnosis, lack of access to trastuzumab, and delay in receiving care due to economic constraints.

Numerous studies have shown that the presence of comorbidities at the time of diagnosis impacts the survival rate among HER2-positive breast cancer patients [18]. Diabetes mellitus was the only comorbidity significantly impacting the mean survival times among early-stage patients in our cohort (log-rank *p* < 0.05). This may be due to diabetic complications potentially limiting the use of adjuvant chemotherapy and radiation [19].

## 5. Conclusion

The 4-year survival rate for both early-stage and advanced-stage HER2-positive breast cancer in our setting is suboptimal when compared to existing outcome data from health care systems where trastuzumab is more widely available. Although a number of factors likely influence this observation, the lack of access to effective HER2-directed therapies are likely a major contributor to these poor outcomes in addition to both the health care system and economic variables.

## Figures and Tables

**Table 1:** Socio-demographic characteristics of HER2-positive breast cancer patients.

Variable	Frequency	Percent
*Age (in years)*		
<60 years	40	80.0
≥60 years	10	20.0
*Marital status*		
Single	7	14.0
Married	35	70.0
Divorced	7	14.0
Widowed	1	2.0
*Educational status*		
Primary	6	12.0
Secondary	19	38.0
Tertiary	25	50.0
*Occupational status*		
Housewife	11	22.0
Government employee	10	20.0
Retired	3	6.0
Merchant	2	4.0
Unemployed	6	12.0
Farmer	4	8.0
Daily laborer	3	6.0
Private employee	11	22.0
*History of substance use*		
Alcohol	7	14.0
Smoking cigarette	1	2.0
None	42	84.0
*Family history of cancer*		
No	25	50.0
Yes	25	50.0

**Table 2:** Clinical characteristics of HER2-positive breast cancer patients.

Variable	Frequency	Percent
*Stage of cancer*		
Stage I	1	2.0
Stage II	17	34.0
Stage III	14	28.0
Stage IV	18	36.0
*Comorbidity*		
Present	22	44.0
Absent	28	56.0
*Number of comorbidities*		
Zero	28	56.0
One	16	32.0
Two	6	12.0
*Any distant metastasis*		
No	32	64.0
Yes	18	36.0
*Type of comorbidity*		
Diabetes mellitus	11	22
Hypertension	7	14
Retroviral disease	7	14
Deep vein thrombosis	2	4
Anemia	1	2

**Table 3:** Treatment regimen administered to the HER2-positive breast cancer patients.

Variable	Frequency	Percent
*Treatment regimen for early-stage disease*		
Radiotherapy	8	25
Chemotherapy	11	34.4
Surgery	10	31.3
Trastuzumab	3	9.3
*Treatment regimen for the incurable disease*		
Radiotherapy	6	33.3
Chemotherapy	11	61.1
Trastuzumab	1	5.6

**Table 4:** Mean survival time estimates among HER2-positive breast cancer patients.

Variable	Mean survival time (months) ± standard error (95% CI)	Log-rank test (*p* value)
*Early-stage curable disease*		
Age (in years)		0.556
<60 years	44.2 ± 2.9 (38.4-49.9)	
≥60 years	39.0 ± 9.9 (19.6-54.3)	
Comorbidity		0.150
Present	38.5 ± 4.2 (30.2-46.8)	
Absent	46.5 ± 3.8 (39.1-53.9)	
Tumor response		0.165
Complete response	57.5 ± 1.8 (54.0-60.9)	
Partial response	49.4 ± 0.8 (47.9-50.9)	
Nonresponse	40.9 ± 5.9 (29.3-52.6)	
Progression of the disease	34.8 ± 5.0 (27.8-50.9)	
Diabetes mellitus		0.032∗
No	45.6 ± 3.1 (39.5-51.7)	
Yes	32.5 ± 6.9 (19.0-45.9)	
*Metastatic disease*		
Age (in years)		
<60 years	9.2 ± 1.1 (6.3-11.3)	0.921
≥60 years	9.1 ± 0.5 (8.3-10.2)	
Comorbidity		
Present	8.9 ± 0.9 (7.2-10.7)	0.840
Absent	9.1 ± 1.4 (6.3-11.9)	
Tumor response		
Nonresponse	8.0 ± 1.0 (6.0-9.9)	0.453
Progression of the disease	9.2 ± 0.9 (7.4-11.1)	
Diabetes mellitus		
No	9.4 ± 1.1 (7.3-11.5)	0.506
Yes	8.2 ± 1.4 (5.5-10.9)	

∗Statistically significant *p* value <0.05.

## Data Availability

The datasets used during the current study are available from the corresponding author on a reasonable request.
